# Bending Toughness and Toughening Mechanism of Polyoxymethylene Fiber-Reinforced Shotcrete

**DOI:** 10.3390/ma19091842

**Published:** 2026-04-30

**Authors:** Shujian Li, Jun You, Menglai Wang, Xiangyun Kong, Guangjin Wang, Yuanting Zhu, Chen Sang, Xiaochuan Xia

**Affiliations:** 1 Yunnan Phosphate Chemical Group Co., Ltd., Kunming 650600, China; lishujiancqu@163.com (S.L.); menglaiwang@163.com (M.W.); 2 Guizhou Institute of Geo-Environment Monitoring, Guiyang 550081, China; zytcug@163.com; 3 Faculty of Architectural Engineering, Kunming University of Science and Technology, Kunming 650500, China; 20120027@kust.edu.cn; 4 Faculty of Land Resources Engineering, Kunming University of Science and Technology, Kunming 650093, China; 5 Faculty of Public Safety and Emergency Management, Kunming University of Science and Technology, Kunming 650093, China; 6 Guizhou Urban and Rural Planning and Design Research Institute Co., Ltd., Guiyang 550081, China; gzyimiyangguang@163.com

**Keywords:** POM fiber, shotcrete, four-point bending test, flexural toughness, reinforcing mechanism

## Abstract

To study the influence of polyoxymethylene (POM) fibers on the mechanical properties of shotcrete for tunnel support, this research conducted four-point bending tests on concrete with different POM fiber dosages (0, 5, 7, and 9 kg/m^3^) and lengths (30 mm, 36 mm, and 42 mm). The mechanical properties are analyzed in terms of failure modes, flexural strength, and the toughness index. The results show that, with the increase fiber length and dosage, the incorporation of POM fibers can enhance the toughness of concrete and significantly improve the flexural performance of shotcrete, with the peak flexural strength increasing by 15.31% to 89.46%. Additionally, through scanning electron microscopy (SEM) image analysis, the reinforcing mechanism of POM fibers is revealed: when shotcrete with POM fibers is subjected to flexural loading, it undergoes four stages: elastic, elastic–plastic, yield, and failure. The addition of POM fibers increases the density and uniformity of concrete, and the flexural strength is indirectly enhanced by increasing frictional energy dissipation through the formation of fiber–matrix interfaces between fibers and concrete. The research findings provide a theoretical basis and design reference for the application of POM fiber-reinforced shotcrete in tunnel support.

## 1. Introduction

As mining operations reach greater depths, tunnel support systems face mounting challenges [1,2]. Under deep mining conditions, roadways surrounding rock deformation intensify, the amount of fractured rock increases, and ground stress rises significantly [3,4,5,6]. Concrete is a key material for roadway support, but conventional concrete has low mechanical strength. Once brittle fracture occurs, it loses load-bearing capacity, compromising the surrounding rock’s stability [7]. To enhance concrete’s mechanical properties, domestic and international scholars have developed ultra-high-performance fiber-reinforced concrete (UHPFRC) by incorporating fibers to achieve high strength and low permeability [8], with applications in construction, roadworks, and mining [9,10,11]. Ashkezari, GD, et al. tested steel fiber-reinforced concrete (SFRC), demonstrating that a 3% steel fiber volume fraction can increase tensile and flexural strength by 228% and 180%, respectively [12]. While SFRC engineering processes are well-established, steel fibers corrode in humid, oxygen-rich environments, making them unsuitable for water-seepage roadways in underground mines [13,14]. These durability concerns have driven increasing research interest in corrosion-resistant synthetic fibers for shotcrete. Recent studies have shown that synthetic macrofibers—particularly polypropylene (PP) and polyvinyl alcohol (PVA)—can significantly improve the post-cracking behavior and residual strength of concrete. For instance, PP macrofibers tested via ASTM C1609 four-point bending have been demonstrated to enhance concrete toughness, with fiber volume, slenderness ratio, and elastic modulus being the key contributing parameters [15]. PVA fibers have been found to effectively improve the flexural strength and crack resistance of concrete, with fiber length playing a more critical role than dosage in enhancing the flexural performance [16]. However, PP fibers, due to their low elastic modulus and hydrophobic nature, tend to exhibit weak bonding with the cement matrix, limiting their reinforcement efficiency in high-strength matrices [17]. For corrosion-resistant fibers like basalt, acrylic, polyvinyl alcohol (PVA), and hybrid fibers, Al-Rousan [18], Hasham M [19], and Yan X [20] discussed the impact of fibers on concrete durability, experimentally determining the optimal fiber length and mix ratio. The study demonstrated that a specific fiber dosage can effectively inhibit concrete cracking and enhance its mechanical strength. However, considering concrete preparation processes and inherent fiber characteristics, basalt fibers, acrylic fibers, and polyvinyl alcohol fibers present practical challenges including poor dispersion characteristics, inadequate enhancement of flexural strength, and high production costs. Polyacetal fiber (POM) is a kind of high performance synthetic fiber. Li [21] compared POM fibers with common synthetic materials such as polypropylene fibers and glass fibers, finding that POM exhibits distinctive characteristics, including alkali resistance, wear resistance, corrosion resistance, and a high modulus, and proposed its application as concrete reinforcement fiber. Numerous studies have confirmed that POM fibers effectively enhance concrete’s mechanical properties while demonstrating superior dispersion properties during mixing, rendering them viable for practical engineering applications to improve concrete performance. Shi, X.S. [22] developed a model for concrete strength degradation and elucidated POM fibers’ reinforcement mechanism through SEM experiments. Zhang, J.C. [23] predicted the strength performance of POM fiber-reinforced concrete using a boundary effect model. Laboratory tests revealed that POM fibers significantly increase concrete’s tensile strength and toughness. Peng et al. [24] found that replacing part of ultra-high-molecular-weight polyethylene fibers with POM fibers can improve the mechanical properties of specimens to a certain extent, while reducing carbon emissions and energy consumption. Wang et al. [25]. found that, owing to its outstanding alkali resistance and durability, POM can effectively enhance the mechanical properties of seawater sea–sand concrete. More recently, Ding et al. [26] investigated the structural use of macro-POM fibers in concrete and compared their flexural performance with macro-PP fibers of the same size and dosage. The results demonstrated that POM fiber-reinforced concrete exhibits similar or superior flexural toughness to PP fiber-reinforced concrete, and the addition of POM fibers significantly enhances the post-cracking toughness.

In summary, POM fibers show superior suitability for underground roadway support compared to other common fibers such as steel fibers. Despite these advances in understanding POM fiber reinforcement, the majority of existing studies have been conducted on laboratory-cast specimens, with little application study in roadway support. Because of its lower density, shotcrete used in roadway support exhibits lower strength and toughness than conventionally cast concrete. Existing studies indicate that stress release occurs perpendicular to the roadway axis after excavation [27,28], and shotcrete support fails primarily through bending. Building on existing research, all specimens in this paper were prepared in strict accordance with relevant specifications and field engineering practice, with the preparation process fully consistent with the actual construction technology of underground roadway support. On this basis, the effects of different dosages and lengths of polyoxymethylene (POM) fibers on the flexural resistance of shotcrete were investigated. The reinforcement mechanism of POM fibers in shotcrete was analyzed through microstructure observations via scanning electron microscopy (SEM). This work provides a valuable reference and practical guidance for similar engineering applications. The test results can authentically reflect the mechanical properties of POM fiber-reinforced shotcrete under service conditions rather than the ideal properties obtained under laboratory-casting conditions.

## 2. Test Materials and Methods

### 2.1. Raw Materials

**Figure 1:**
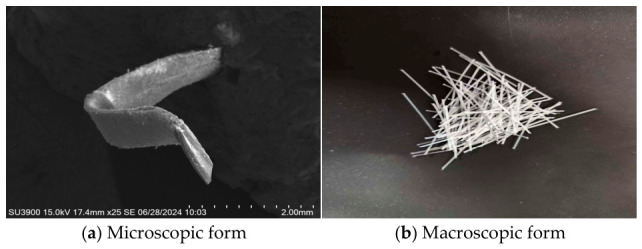
POM fiber morphology.

To ensure the consistency of test results with actual engineering applications, the fiber reinforcement used was polyoxymethylene (POM) from Chongqing Yuntianhua Tianju New Materials Co. (Chongqing, China), and the cement was PO42.5 grade ordinary Portland cement. Coarse aggregates were selected as continuously graded pebbles or crushed stone with a particle size ≤ 12 mm. Fine aggregates were Zone II medium sand, having a fineness modulus of 2.5–3.2. The fibers had a flat cross-section, and the specific material parameters are listed in Table 1. This study examined POM fibers at three designated lengths: 30 mm, 36 mm, and 42 mm. The microscopic and macroscopic structures of the fibers are shown in Figure 1. The detailed mix proportion of fiber concrete is shown in Table 2.

### 2.2. Specimen Production

**Figure 2:**
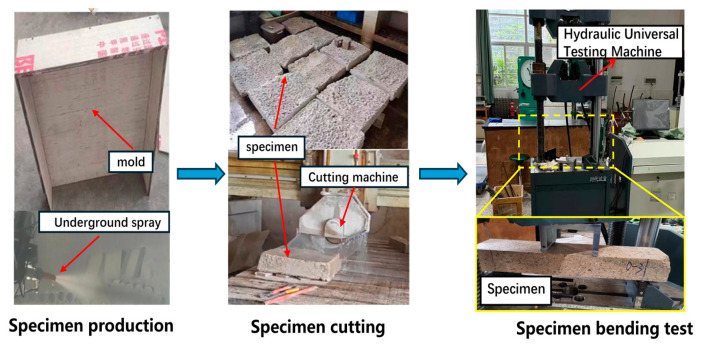
POM fiber shotcrete preparation procedure for bending test.

To fully investigate the flexural properties of shotcrete, all test specimens in this study were cut from large-scale integrally cast shotcrete slabs. Specimen preparation was carried out in strict accordance with GB 50086-2015 [29]. During testing, the formwork for the large shotcrete slab was placed leaning against a wall at an inclination angle of approximately 80°. After the spraying rate of the shotcrete equipment stabilized, concrete was applied layer by layer from the bottom upward to fill the formwork. Upon completion of spraying, the slab was cured in a humid environment for 1 day before formwork removal, and then immediately transported to a standard curing room for an additional 7 days of curing. After curing, the large shotcrete slab was cut into small beam specimens with dimensions of 600 mm × 125 mm × 75 mm for flexural strength testing. Following specimen preparation, they were further cured in the standard curing room until the age of 28 days. The specimen preparation process is illustrated in Figure 2.

### 2.3. Test Methods

**Figure 3:**
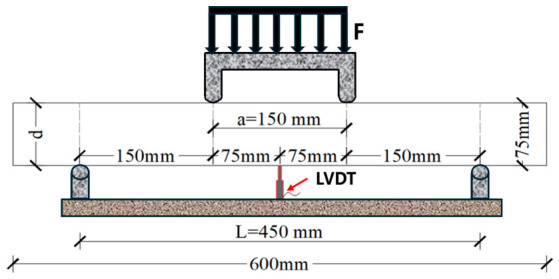
Schematic diagram of flexural strength test.

The flexural strength test was performed according to the CECS 13:2009 [30]. In accordance with the specification, the failure criterion of the specimen was defined as follows: the mid-span deflection of the specimen reached 1/150 of the span (i.e., 3 mm in this study), or the specimen underwent complete fracture and lost its load-bearing capacity entirely. A WAW-300D electro-hydraulic universal testing machine was used for four-point loading. After extraction from the curing chamber, we placed the specimens on the testing machine supports and adjusted their position and orientation. A preload was applied to normalize stress distribution across the specimen surface. An LVDT displacement sensor was installed at the mid-span of the specimen. During loading, and the mid-span strain rate was maintained at 0.2 mm/min to 0.3 mm/min, respectively, until specimen failure occurred. Then, the load magnitude, failure mode, and specimen deflection were recorded. The test loading configuration is shown in Figure 3, and the flexural strength calculation formula is given in Equation (1) as follows.

(1) $$f_{c}=\frac{P_{0.1}\times L}{b\times d^{2}}$$

where f_c_ is the standard value of the flexural strength of concrete (MPa). P_0.1_ is the load corresponding to the intersection of the deflection value and the deflection curve of the straight-line segment, with a deflection value of 0.1 mm translation in the load deflection curve (N). L is the width between instrument stands.

### 2.4. Microscopic Performance Testing

Microstructural analysis was carried out using scanning electron microscopy (SEM) by taking a small piece of the specimen after 28 d of standard curing from the center of the broken specimen at the end of the test; then, the sample was adhered to the conductive adhesive on the mounting pads and placed in an ion sputtering coater for gold plating. After surface gold plating, the samples were placed in a vacuum chamber of SEM for observation, and a scanning electron microscope was used to capture the microstructure of the cement matrix, the surface morphology of the POM fibers, as well as the embedded morphology between the fibers and the matrix.

## 3. Experimental Results and Analyses

### 3.1. Flexural Strength Analysis

**Figure 4:**
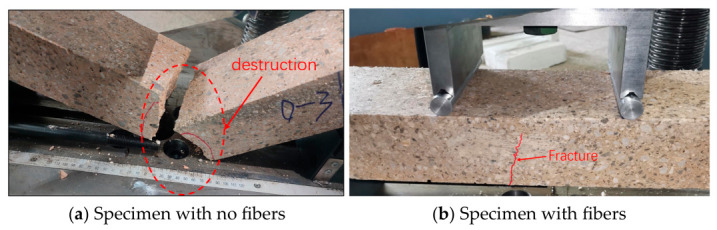
Damage of shotcrete flexural specimens.

Through the four-point bending test of sprayed concrete beam specimens with a span of 450 mm, the ordinary sprayed concrete specimens of the POM0-0 group suddenly broke from the middle of the specimen with a ringing sound after the peak load was reached during the test, showing obvious brittle damage. Meanwhile, for the POM fiber-sprayed concrete specimens, cracks began to appear on the outer surface of the specimen with the increase in load, but the generation rate was slow, and when the load reached the peak, cracks appeared on the outer surface of the specimen with a rapid decrease in load, but the specimen did not show brittle fracture. When the load reaches the peak value, cracks appear on the outer surface of the specimen, and the load decreases rapidly, but the specimen does not show brittle fracture, and after continuing to apply load to it for a period of time, the fibers will be pulled out from the concrete or pulled off. Among them, the damage of four-point bending test specimens is shown in Figure 4.

**Figure 5:**
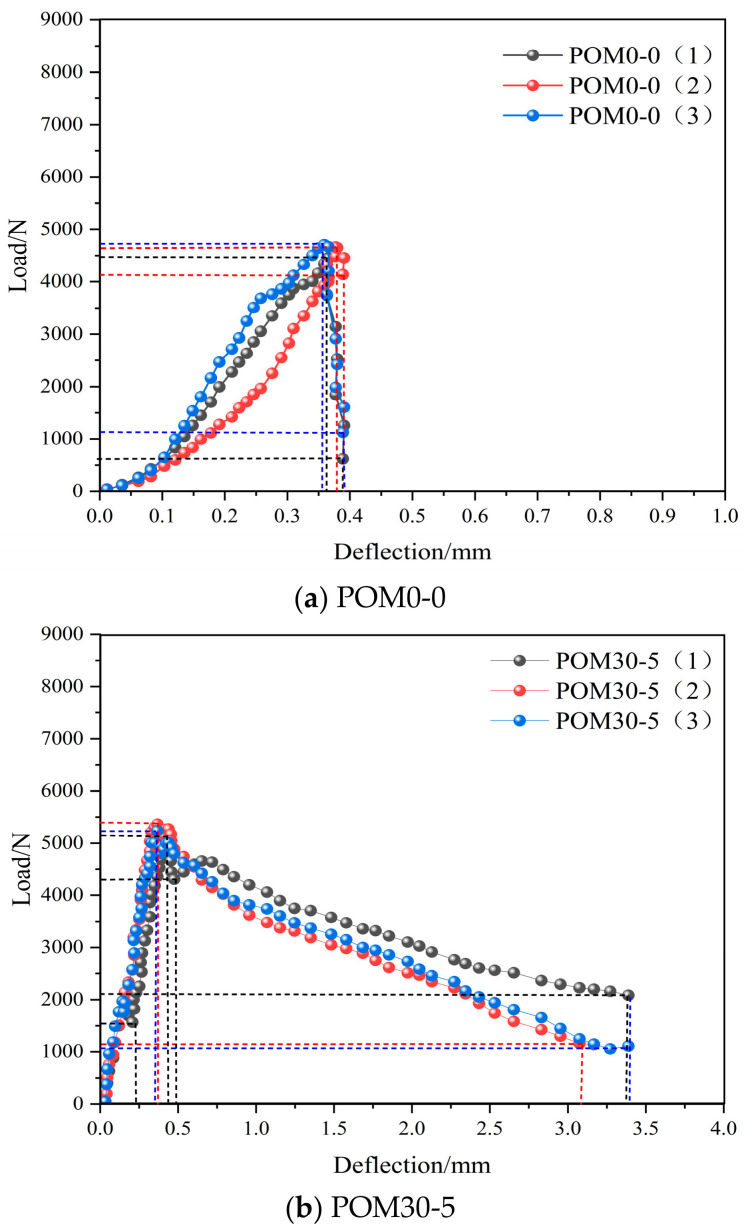

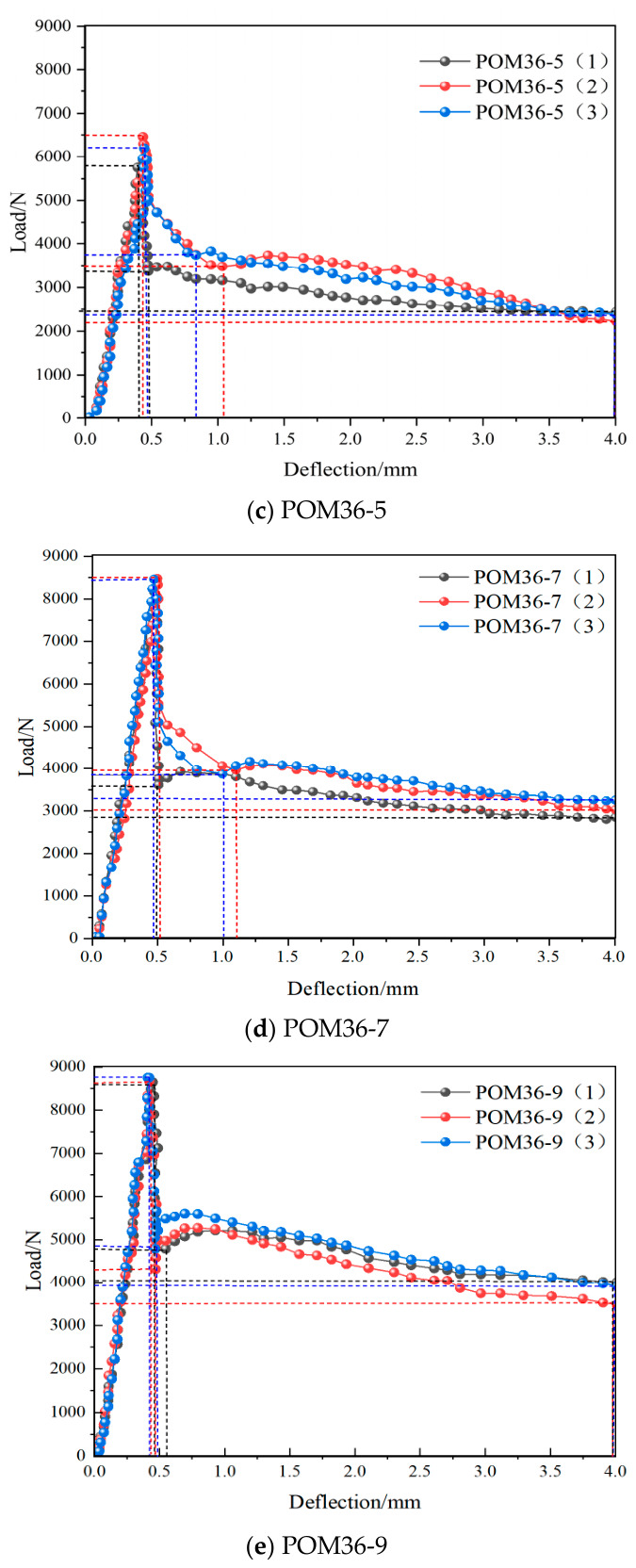

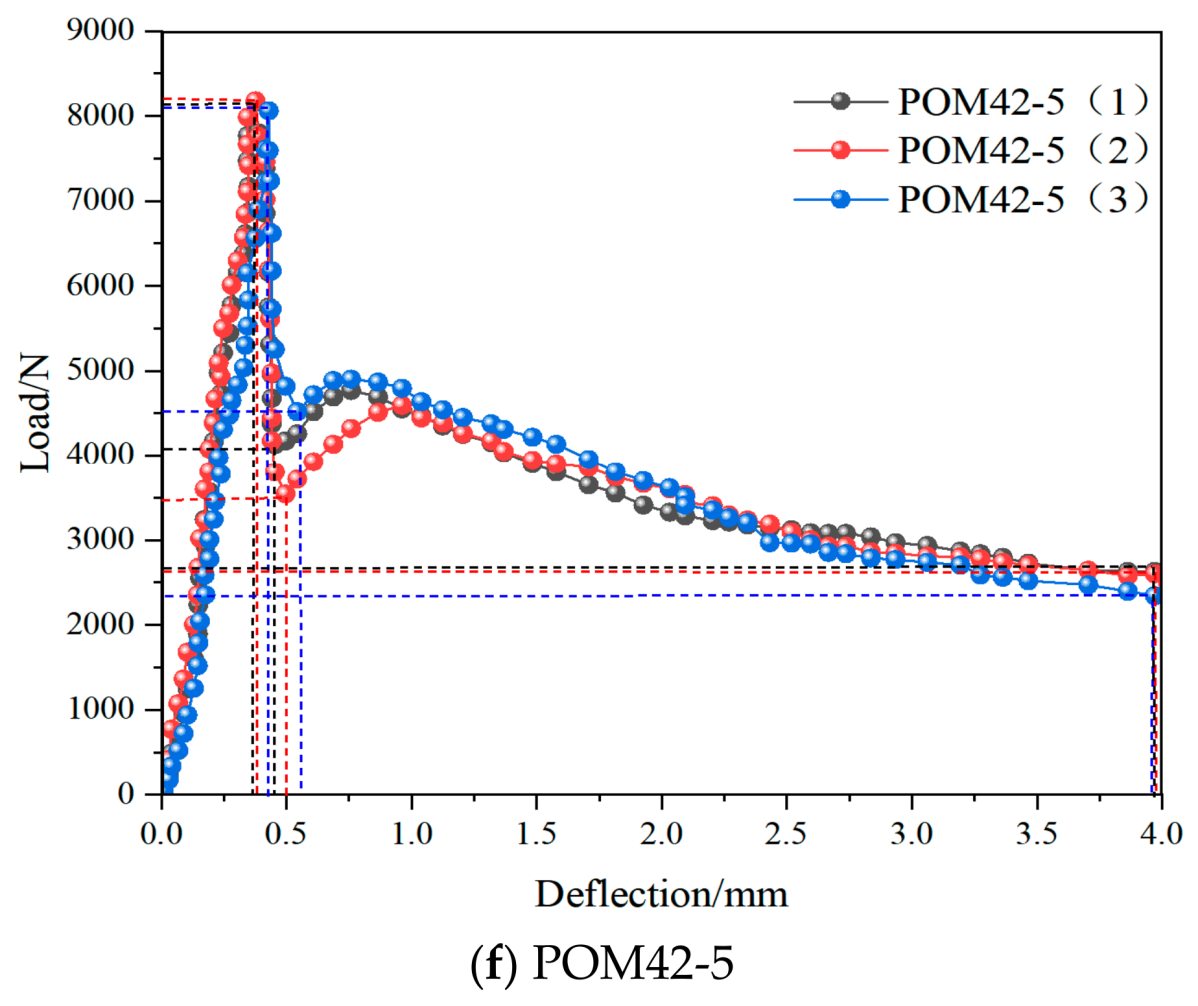
Load–deflection curve of flexural specimen.

Figure 5 presents the load–deflection curves of all specimen groups obtained from four-point bending tests on shotcrete specimens. In Figure 5a, the plain shotcrete specimen suffered brittle fracture directly during the four-point bending test, leading to a measured mid-span deflection of less than 0.5 mm. In contrast, the POM fiber-reinforced shotcrete specimens shown in Figure 5b–f can maintain a certain load-bearing capacity, even after reaching the peak failure load due to the bridging effect of fibers. The resulting curves exhibit certain ductility, and the measured deflection meets the specification [30] requirements.

In Figure 5, the dotted lines are only used to mark the abscissa and ordinate values of the corresponding points on the curve, with no other special meanings. From Figure 5a–f, it can be observed that the control group, POM0-0 ordinary sprayed concrete specimens, shows a rapid load drop after reaching peak capacity, demonstrating clear brittle behavior. In contrast, POM fiber-reinforced concrete initially shows a linear deflection increase proportional to the load. After reaching peak load, the load drops instantaneously due to specimen surface failure. However, owing to the fibers’ bridging effect, the load demonstrates a recovery trend, and the load–deflection curve exhibits secondary peak loads of varying magnitudes. As the fiber dosage and length increased, these secondary peaks become more conspicuous. This indicates that POM fiber incorporation enables the concrete to demonstrate better ductility and toughness, effectively enhancing both toughness and crack resistance.

In this study, the mean value (μ) and standard deviation (σ) of the flexural strength of each group of specimens were calculated according to Equation (2). The test results indicate that each group exhibits low data dispersion, demonstrating good reliability of the experimental data. The detailed calculation results are presented in Table 3.

(2) $$\sigma =\sqrt{\frac{1}{N}\sum_{i=1}^{N}(x_{i}-\mu {)}^{2}}$$

where x_i_ is the flexural strength of each specimen; N is the total number of flexural strength data (three in total); and μ represents the mean value.

**Figure 6:**
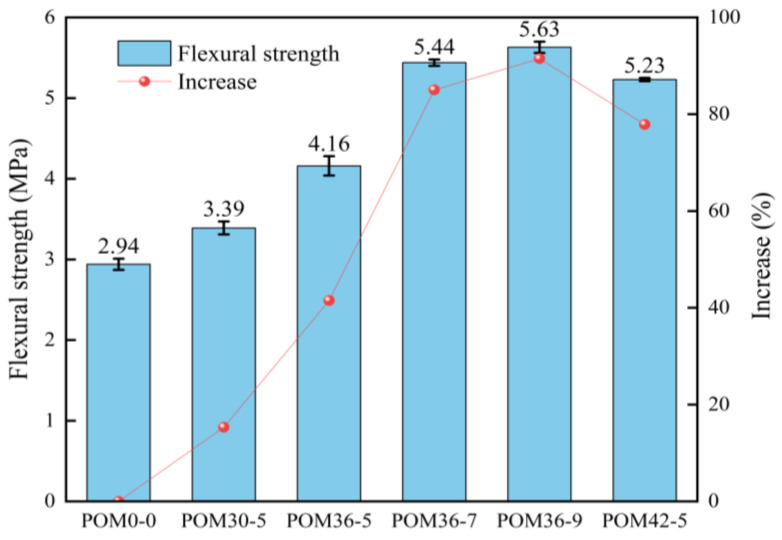
Flexural strength and reinforcement of fiber shotcrete.

According to Equation (1), the flexural strength is calculated from the load–deflection curves in Figure 5, producing the flexural strength values and the performance enhancement from POM fibers on the flexural properties of sprayed concrete, as shown in Figure 6.

As shown in Figure 6, the flexural strength of ordinary shotcrete is 2.94 MPa. With POM fiber incorporation, the flexural strength of shotcrete shows enhancement. Among all test groups, the POM36-9 group demonstrates optimal enhancement in flexural strength, followed by POM36-7, in that order. When the fiber dosage is held constant at 5 kg/m^3^, the increase in flexural strength of shotcrete increases proportionally with the fiber length, showing improvements of 15.31%, 39.46%, and 77.55%, correspondingly. When the fiber length is held constant at 36 mm, the flexural strength increases with the increased fiber content, showing improvements of 39.46%, 83.67%, and 89.46%, respectively.

The core mechanism underlying the enhancement of shotcrete flexural strength by POM fibers lies in the increased effective fiber bridging area at crack surfaces and the improved fiber–matrix interfacial bonding properties. At a constant fiber dosage of 5 kg/m^3^, increasing the fiber length from 30 mm to 36 mm extends the anchorage length of fibers within the matrix and reduces the likelihood of premature fiber pull-out prior to attaining their ultimate tensile strength, which aligns with the findings of Del Savio et al. [15]. Nevertheless, further increasing the fiber length to 42 mm results in a decline in flexural strength. For a fixed fiber length of 36 mm, increasing the dosage from 5 kg/m^3^ to 7 kg/m^3^ yields a substantial 44.21% increase in flexural strength, whereas a further increase from 7 kg/m^3^ to 9 kg/m^3^ only produces a marginal 5.79% improvement. Both the strength reduction induced by excessively long fibers and the diminishing enhancement effect caused by excessive fiber content can be attributed to fiber agglomeration within the concrete matrix. This agglomeration introduces new weak interfaces that partially counteract the beneficial reinforcing effects, a phenomenon that has also been documented in previous studies [23,31].

POM fibers can remarkably enhance the flexural performance of shotcrete, converting its failure mode from brittle fracture to ductile failure. The flexural strength increases monotonically with the increase in fiber content, while it first rises and then falls with the increase in fiber length. Among all groups, the POM36-9 group achieves the best comprehensive toughening and strengthening effect.

### 3.2. Flexural Toughness Analysis

Toughness is a key parameter for evaluating a material’s resistance to seismic, blast, and impact load. To further assess the bending performance of shotcrete with POM fibers and study the post-cracking fiber reinforcement effect, this paper evaluates specimens’ bending toughness through energy absorption values and fracture energy analysis.

(1) Energy Absorption Value

Energy absorption value represents the energy absorbed by the specimen when withstanding bending loads until failure. A higher value demonstrates better bending toughness of the shotcrete specimen, providing better resistance against dynamic loads including seismic and impact forces.

**Figure 7:**
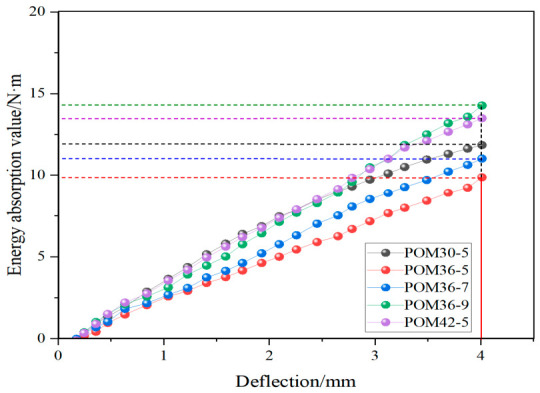
Bending energy absorption curve.

By performing multi-degree polynomial fitting on the four-point bending load–deflection curves of each specimen group in Figure 5 and applying numerical integration of the fitted curves, the bending energy absorption curves for different mixture proportions were obtained to represent the toughness and energy absorption capacity of the fiber-reinforced concrete. The results are as illustrated in Figure 7.

**Figure 8:**
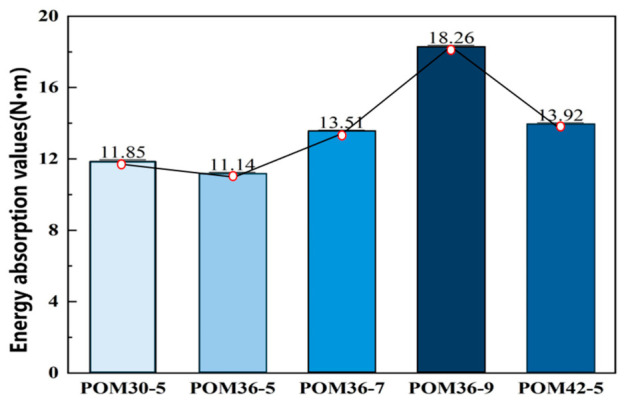
Energy absorption values of fiber concrete beams.

The energy absorption values of fiber-reinforced sprayed concrete beams with different mix ratios shown in Figure 8 were obtained by statistically analyzing the energy absorption curves in Figure 7. The figure shows that the POM36-9 group had the largest area under its load–deflection curve, indicating the highest energy absorption value of 18.26, while the POM36-5 group had the smallest value of 11.14. When the fiber length of 36 mm was used as the quantitative value, the energy absorption value increases with the increase in the dosage, in which the POM36-9 group was the largest. When the fiber dosage of 5 kg/m^3^ is taken as the quantitative value, the energy absorption values show a decreasing and then increasing trend, with POM42-5 being the largest and POM36-5 being the smallest. As the fiber content increases, a denser three-dimensional (3D) fiber network structure is formed within the matrix, while the increase in fiber length enhances the anchoring effect between the fibers and the matrix. In the four-point bending test, a greater number of fibers can thus effectively exert their reinforcing effect: they dissipate external energy via mechanisms including fiber pull-out and interfacial slip and markedly boost the interfacial frictional energy dissipation, leading to a continuous increase in the overall energy absorption capacity of the specimens with the rising fiber content [32,33,34]. Consistent with the findings presented in Section 3.1, fiber agglomeration induced by excessively long fibers or excessive fiber content impairs the reinforcing efficiency of fibers within the concrete matrix, resulting in reduced energy absorption and fracture energy or a diminishing enhancement magnitude.

(2) Fracture Energy

Fracture energy is an important parameter for evaluating material toughness and resistance to crack propagation, representing the energy consumed per unit area during crack propagation in concrete beams. By comparing the fracture energy of POM fiber-reinforced sprayed concrete with different mix ratios, the enhancement effect of fibers on fracture resistance can be analyzed. Higher values indicate more effective fiber bridging and a greater delay in crack development.

The fracture energy of POM fiber-reinforced sprayed concrete four-point bending beam specimens is calculated using Equation (3):

(3) $$G_{F}=\frac{W_{f}}{A}=\frac{w_{0}+\frac{1}{2}mg\delta_{max}}{\left(h-a_{0}\right)t}$$

where w_0_ is the area enclosed by the load–deflection curve, δ_max_ is the maximum deflection at mid-span at the end of the test (mm), h is the specimen height (mm), a_0_ is the length of preformed seam (mm),t is the specimen width (mm), m is the mass of the specimen within the span between the fixed and movable support (kg), and g is the gravitational acceleration (9.8 N/kg).

**Figure 9:**
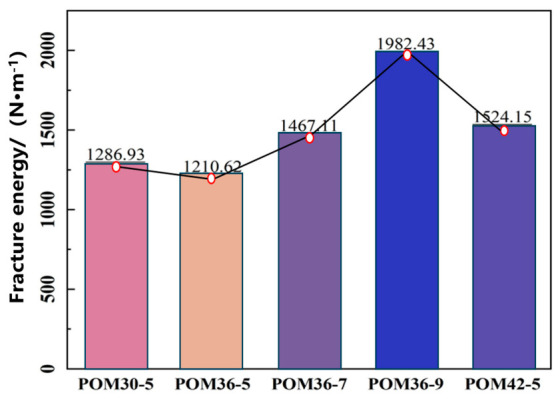
Fracture energy of fiber concrete beams.

Applying Equation (3) to calculate the fracture energy of fiber-reinforced concrete four-point bending beams with different mix ratios yields the results shown in Figure 9. The results demonstrate that the POM36-9 group generally has the highest fracture energy at 1982.43, while the POM36-5 group has the lowest at 1210.62. With the fiber length held constant at 36 mm, the fracture energy increases with the higher fiber content.

The flexural toughness and post-crack reinforcement effect of POM fiber-reinforced shotcrete were evaluated with the energy absorption value and fracture energy as the core indicators. The results show that a certain range of fiber dosage and length can significantly improve the energy absorption capacity and crack resistance. Among all test groups, the POM36-9 group exhibited the optimal energy absorption value and fracture energy. When the fiber length was 36 mm, the flexural toughness increased continuously with the increase in fiber dosage. When the fiber dosage was 5 kg/m^3^, the toughness first decreased and then increased with the increase in fiber length, and the POM42-5 group showed a better performance.

In summary, POM fibers can significantly enhance the post-crack energy dissipation and crack resistance of shotcrete, thereby improving its flexural toughness.

## 4. The Microscopic Study of Flexural Damage in POM Fiber Concrete

**Figure 10:**
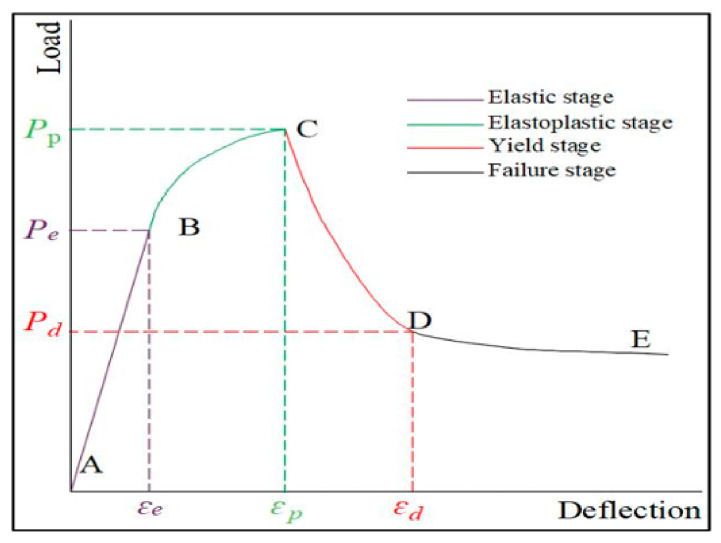
Load–deflection curve of POM fiber concrete.

Plain shotcrete exhibits linear load–deflection behavior and brittle failure after peak load. For POM fiber-reinforced shotcrete, the load–deflection curve can be divided into four deformation stages according to the test results. In this section, SEM observations are used to show the micro-morphology of POM fibers and the matrix corresponding to each stage so as to reveal the influence of POM fibers on the deformation and failure process of shotcrete. The schematic diagram of the load–deflection curve is shown in Figure 10, and the characteristics of each stage are described as follows.

**Figure 11:**
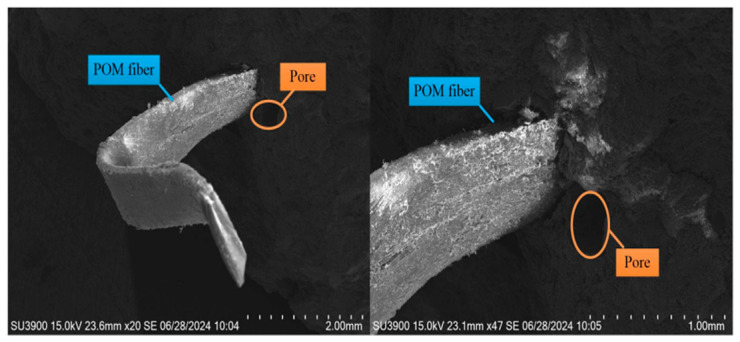
Microstructure of POM fibers and concrete in elastic phase.

**Figure 12:**
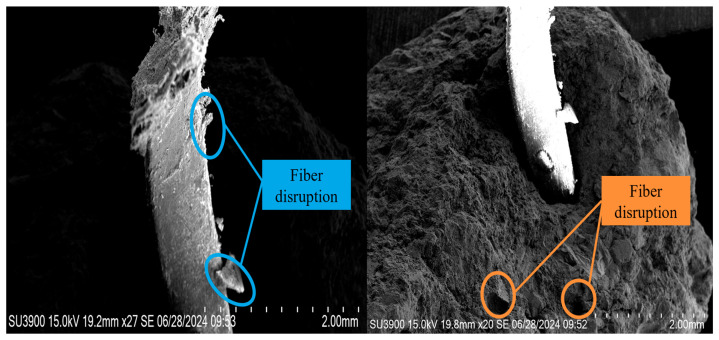
Microstructure of POM fibers and concrete in elastic–plastic phase.

**Figure 13:**
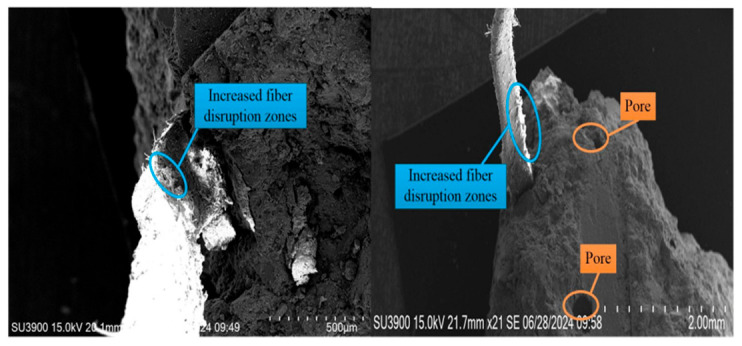
Microstructure of POM fibers and concrete in yield phase.

**Figure 14:**
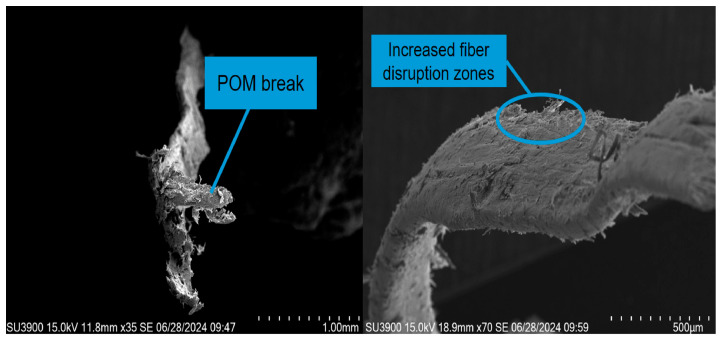
Microstructure of POM fibers and concrete in destruction phase.

(1) Elastic stage (AB): In the initial loading stage, POM fiber-reinforced concrete undergoes bending deformation due to the load. Deflection increases linearly with the load until the elastic limit (Pe) is exceeded, at which point cracks appear at the bottom of the concrete. The POM fibers remain intact, and both concrete and fibers jointly bear the external load. The microscopic morphology of the concrete in the elastic stage is shown in Figure 11.

(2) Elastic–plastic stage (BC): After cracking, the concrete enters the elastic–plastic stage. Load and deflection continue to increase, but the slope of the deflection–load curve is smaller than in the elastic stage. The deflection rate accelerates until the peak load (Pp) is reached, and the specimen undergoes plastic yielding. The external load is primarily borne by the POM fibers, which begin to fail at this stage. The microscopic morphology of the concrete in this stage is shown in Figure 12.

(3) Yielding stage (CD): The load on the specimen drops rapidly after the peak load, while the deflection continues to increase slowly. The bottom cracks extend upward, and the concrete loses its load-bearing capacity. The POM fibers bear the entire external load, suffering extensive damage. The microscopic morphology of the concrete in the yielding stage is shown in Figure 13.

(4) Failure stage (DE): The load exceeds the bearing limit of both the concrete and POM fibers. The rates of crack propagation and deflection increase rapidly, and both fibers and concrete lose their load-bearing capacity, leading to specimen failure. The POM fibers fracture, and the microscopic morphology of the concrete in this stage is shown in Figure 14.

The load–deflection curve of the POM fiber-reinforced shotcrete under flexural load is divided into four stages: elastic stage, elastoplastic stage, yield stage, and failure stage. SEM observations show that POM fibers convert brittle fractures into ductile failures by dissipating energy through interface friction and improving the compactness of the matrix, which reveals the micro-scale toughening mechanism.

In summary, the SEM observations presented in this section capture the morphological changes in POM fibers and the surrounding cement matrix across the four loading stages, offering microstructural evidence that aligns with the load–deflection behavior discussed in Section 3. While the flexural performance of the shotcrete has been assessed quantitatively through flexural strength, energy absorption values, and fracture energy, a more detailed microstructural characterization—covering aspects such as the fiber–matrix interfacial bonding strength, pore structure parameters (e.g., via mercury intrusion porosimetry), phase identification by X-ray diffraction, and elemental composition or chemical bonding across the interface (e.g., via EDS or FTIR)—remains to be explored.

## 5. Conclusions

(1) In the four-point bending test of concrete beams, ordinary shotcrete specimens fracture mid-span after reaching peak load, whereas fiber-reinforced specimens exhibit excellent toughness. The bending strength of concrete increases with the fiber length and dosage. At a fixed fiber dosage of 5 kg/m^3^, the increases are 15.31%, 39.46%, and 77.55%, respectively. At a fixed fiber length of 36 mm, the increases are 39.46%, 83.67%, and 89.46%, respectively. The calculated energy absorption and fracture energy of the POM36-9 group are the highest among all mixes, reaching 18.26 and 1982.43, respectively.
(2) POM fibers improve the bending performance of shotcrete. After incorporating POM fibers, the failure mode shifts from brittle to ductile, effectively inhibiting crack propagation. Experimental results show that the POM fiber dosage significantly affects the bending performance. After concrete cracking, the tensile properties of POM fibers and fiber–matrix friction continue to bear the external load. The optimal improvement is observed at a POM fiber dosage of 7 kg/m^3^, with the most pronounced effect.
(3) The reinforcing mechanism of POM fibers operates at three levels: Firstly, high-speed spraying enhances concrete compactness and uniformity. Secondly, fiber–matrix interfacial friction absorbs energy. Finally, a 3D fiber network forms to effectively transfer and distribute loads. This multi-scale synergy results in enhanced mechanical properties and failure characteristics. SEM reveals that POM fiber-reinforced shotcrete initially undergoes elastic deformation under the bending load with a linear deflection curve until cracking occurs, transitioning to the elastic–plastic stage. In this stage, deflection increases significantly until the peak load is reached, entering the yielding stage. The concrete retains some bending capacity, but the load drops rapidly while deflection continues to increase. Upon reaching residual flexural strength, the failure stage begins, where deflection increases rapidly as the load declines slowly, and the concrete loses its load-bearing capacity.

The results obtained here provide a valuable basis for future research on design parameter studies for tunnel shotcrete support.

## Figures and Tables

**Table 1:** Physical and mechanical properties of polyformaldehyde fibers.

Project	Unit	Parameter
density	g/cm^3^	1.41
equivalent diameter	mm	0.44
modulus of elasticity	GPa	≥7
tensile strength	MPa	≥750
elongation at break	%	≤20

**Table 2:** Fiber-reinforced concrete mix design.

Group	Cement (kg·m^−3^)	Aggregate (kg·m^−3^)	Water (kg·m^−3^)	Length (mm)	Dosage (kg·m^−3^)
POM0-0	300	1260	185	-	-
POM30-5				30	5
POM36-5				36	5
POM36-7				36	7
POM36-9				36	9
POM42-5				42	5

**Table 3:** Statistical values of flexural strength of fiber-reinforced concrete.

Group	*f_f_*/MPa			Group	*f_f_*/MPa		
	*x_i_*	*μ*	*σ*		*x_i_*	*μ*	*σ*
POM0-0	2.84	2.94	0.07	POM36-7	5.34	5.40	0.04
	3.00				5.43		
	2.99				5.43		
POM30-5	3.28	3.39	0.08	POM36-9	5.48	5.57	0.07
	3.47				5.59		
	3.42				5.64		
POM36-5	3.94	4.10	0.12	P5L42	5.20	5.22	0.02
	4.17				5.22		
	4.20				5.25		

## Data Availability

The original contributions presented in this study are included in the article. Further inquiries can be directed to the corresponding authors.
